# CLP1 is a Prognosis-Related Biomarker and Correlates With Immune Infiltrates in Rheumatoid Arthritis

**DOI:** 10.3389/fphar.2022.827215

**Published:** 2022-06-01

**Authors:** Zhenyu Zhao, Shaojie He, Sheng Tang, Xiaofeng Lai, Jie Ren, XinCheng Yu, Jinhua Lin, Mohan Wang, Mariya M. El Akkawi, Shan Zeng, Dingsheng Zha

**Affiliations:** ^1^ Department of Orthopaedics, The First Affiliated Hospital, Jinan University, Guangzhou, China; ^2^ Department of Rheumatology, The First Affiliated Hospital, Jinan University, Guangzhou, China; ^3^ School of Basic Medicine and Public Health, Jinan University, Guangzhou, China; ^4^ Department of Plastic and Reconstructive Surgery, Zhujiang Hospital of Southern Medical University, Guangzhou, China

**Keywords:** rheumatoid arthritis, RNA modification, diagnostic biomarkers, gene expression profile data, CLP1

## Abstract

Rheumatoid arthritis (RA) is a chronic, heterogeneous autoimmune disease with a high disability rate that seriously affects society and individuals. However, there is a lack of effective and reliable diagnostic markers and therapeutic targets. In this study, we identified diagnostic markers of RA based on RNA modification and explored its role as well as degree of immune cell infiltration. We used the gene expression profile data of three synovial tissues (GSE55235, GSE55457, GSE77298) from the Gene Expression Omnibus (GEO) database and the gene of 5 RNA modification genes (including m6A, m1A, m5C, APA, A-1), combined with cluster analysis, identified four RNA modifiers closely related to RA (YTHDC1, LRPPRC, NOP2, and CLP1) and five immune cells namely T cell CD8, CD4 memory resting, T cells regulatory (Tregs) Macrophages M0, and Neutrophils. Based on the LASSO regression algorithm, hub genes and immune cell prediction models were established respectively in RA and a nomogram based on the immune cell model was built. Around 4 key RNA modification regulator genes, miRNA-mRNA, mRNA-TF networks have been established, and GSEA-GO, KEGG-GSEA enrichment analysis has been carried out. Finally, CLP1 was established as an effective RA diagnostic marker, and was highly positively correlated with T cells follicular helper (Tfh) infiltration. On the other hand, highly negatively correlated with the expression of mast cells. In short, CLP1 may play a non-negligible role in the onset and development of RA by altering immune cell infiltration, and it is predicted to represent a novel target for RA clinical diagnosis and therapy.

## Introduction

Rheumatoid arthritis (RA) is a chronic autoimmune disease characterized by inflammation of the connective tissue. Statistics show that the incidence of RA in the population is about 1%, which is a common chronic disease (Firestein and McInnes 2017). RA mainly affects joints and joint synovial cartilage. As the disease progresses, the damage of both will become more severe (Littlejohn and Monrad 2018). Cartilage destruction, bone erosion, and other symptoms will gradually occur, eventually leading to irreversible limb deformity and disability, bringing a serious burden to society or individuals (Smolen et al., 2016). It is worth noting that there is a huge gap between early and late symptoms of RA. In the early stage, it is usually accompanied by mild systemic symptoms, including fatigue and morning stiffness, while in the late stage, it is often accompanied by severe multi-system autoimmune diseases, such as pulmonary interstitial disease, multiple vasculitis, etc. (Brzustewicz et al., 2017). Therefore, timely and accurate early diagnosis of RA is particularly crucial. In the current clinical management, rheumatoid factor (RF) and anti-citrullinated protein antibody (ACPA) have become important serum markers for the diagnosis of RA. However, both are often negative in early RA, and the diagnostic validity cannot meet clinical needs (Wu C. Y. et al., 2021). Some new biomarkers such as Fibrinogen-Like Protein 1 (FGL1) (Liu et al., 2020), collagen triple helix repeat containing 1 (CTHRC1) (Myngbay et al., 2019), etc. have potential diagnostic markers, but large-scale clinical validation has not yet been carried out, and there is still a long way to go in clinical services. As a result, finding novel biomarkers that can accurately identify the diagnosis and prognosis of RA is critical.

As gene sequencing technology advances, the importance of RNA modification in the onset and progression of many illnesses has been increasingly elucidated. (Djebali et al., 2012). Cancer, cardiovascular disorders, genetic birth abnormalities, metabolic diseases, neurological diseases, and mitochondrial-related defects are all linked to RNA-modifying enzyme mutations. (Jonkhout et al., 2017). N6-methyladenosine (m6A), N1-methyladenosine (m1A), 5-methylcytidine (5mC), and other RNA modifications are common. Previous research has found that certain RNA changes play a vital role in the etiology and development of RA, and that they might be used as a therapeutic target for the disease. (Wu S. et al., 2021), but similar studies are insufficient, and further in-depth exploration is still needed.

In addition, as an autoimmune disease, the infiltration of immune cells is a key part of the pathogenesis and progression of RA. Studies have suggested that the interactions between a variety of immune cells, inflammatory factors, and various cellular chemokines, including T cells, B cells, macrophages, and NK cells, are involved in the pathological process of RA (Weyand and Goronzy 2021). In addition, it has been suggested that the infiltration of immune cells may be related to the modification of RNA (Gao et al., 2020).

tIn this study, We used gene expression profiling data for three synovial tissues (GSE55235, GSE55457, GSE77298) and five RNA-modifying genes (including m6A, m1A, m5C, APA, A-1) in the Gene Expression Omnibus (GEO) database to obtain the original data information. Combined with cluster analysis, four RNA modifiers (YTHDC1, LRPPRC, NOP2, and CLP1) and five immune cells (T cells CD8, CD4 memory resting, T cell regulatory (Tregs) macrophages M0 and medium neutrophils) were closely related to the process of RA. Among them, T cell subsets, macrophages, and mast cells are considered the three most critical immune infiltrating cells in the process of RA. Hub genes/immune cell prediction models in RA based on the LASSO regression algorithm were established respectively. Simultaneously a nomogram based on the immune cell model was established. Then, we perform molecular typing based on the expression patterns of RNA modification regulators and analyzed protein interaction networks to construct miRNA/transcription factor (TF)-mRNA modifier interaction networks. Finally, we consider CLP1 the most potent RA diagnostic marker among the four key RNA modifiers, and immune correlation analysis suggests that it is highly positively correlated with T-cell follicular helper (Tfh) infiltration. CLP1 is positively correlated with the infiltration of immune cells such as B cells naïve, eosinophils, monocytes, dendritic cells activated, plasma cells, macrophages, T cells CD8^+^ and Tfh, while negatively correlated with the degree of infiltration of mast cells, NK cells activated and T cells CD4 memory.

## Article Types

The article types is Original Reasearch.

## Materals and Methods

### Data and Differentially Expressed Genes Acquirement

The gene expression profile data of synovial tissues GSE55235 (Woetzel et al., 2014), GSE55457 (Woetzel et al., 2014), and GSE77298 (Broeren et al., 2016) were retrieved using the R package in GEOquery (Davis and Meltzer 2007) by accessing the GEO database (Barrett et al., 2007), and the gene expression groups were merged and split into 39 synovial tissue of rheumatoid arthritis and 27 normal synovial tissues. We preprocessed the downloaded expression matrix, including data background adjustment, normalization, and summarization. The 5 gene sets of RNA modification writer genes (including m6A,m1A,m5C, APA, A-1) were obtained by Chen H et al.(Chen et al., 2021) and Cong P et al.(Cong et al., 2021): In order to analyze the changes in the expression values of 5 RNA modification writer genes in the synovial tissues of rheumatoid arthritis relative to the normal tissues, we further screened the differentially expressed genes in RA and normal synovium. DEG is recognized and integrated by the Limma package (Ritchie et al., 2015) and FunRich software (Pathan et al., 2015) in R, we set the genes with logFC>1 and adjPvalue<0.05 as up-regulated genes. The genes with logFC<1 and adjPvalue<0.05 as down-regulated genes. Visualization of chromosomal localization of RNA modification writer genes was done using the R circos package (An et al., 2015).

### Immune Infiltration Analysis

CIBERSORT is a deconvolution technique based on the premise of linear support vector regression for immune cell subtype expression matrices, CIBERSORT was initially used for the analysis of tumor microenvironment (TME) and is now being increasingly applied in the characteristic analysis of immune infiltration in non-tumor tissues (Ge et al., 2021). The synovial tissue of rheumatoid arthritis is composed of various immune and inflammatory cells, interstitial tissue, cytokines, and chemokines, which is an integrated loading system. The infiltration analysis of immune cells has an important guiding role in disease research and prognosis prediction. RNA-Seq data were used to assess the infiltration of immune cells in synovial tissues of rheumatoid arthritis and normal tissues (Newman et al., 2019). CIBERSORT algorithm was used to analyze the immune infiltration between the rheumatoid and normal tissues, to identify the immune cells that were differentially enriched between the diseased and normal tissues in the two sets of data, to calculate the Pearson correlation coefficient between the expression level of key genes and immune cells, and to evaluate the relationship between the key genes and the immune infiltration level.

### Construction of Prediction Model

Immune cells of significantly different infiltration levels and significantly different genes between the rheumatoid and normal synovial tissues were used to construct predictive models. LASSO regression analysis in the R glmnet package (Friedman et al., 2010) was performed on the training set. The LASSO approach can reduce the dimensionality of high-latitude data, allowing a model with fewer variables to explain the data’s features (Gui and Li 2005). To avoid overfitting the model design of the training set, tenfold cross validation is performed. Finally, the regression coefficients generated using LASSO regression analysis are used to create a scoring system. We split all RA patients into high-risk and low-risk groups using the R package “SurvMiner” threshold, and then used principal component analysis (PCA) to see how well the model could identify overall survival outcome events.

### RNA Modification Factor Molecular Typing

We use the R-packet “ConsensusClusterPlus” (Wilkerson and Hayes 2010) for clustering classification, and the samples were divided into different groups by expression of RNA modification factor. The parameter is set to repeat 50 times (reps = 50) and the resampling rate is 80% (pItem = 0.8). Principal component analysis (PCA) was done on the expression levels of all genes to determine the success of grouping, and the results were shown using the “pheatmap” program.

### Construction of Protein-Protein Interaction (PPI) Network

Individual proteins interact with one another in protein interaction networks, which involves various aspects of biological signal transmission, gene expression management, energy and chemical metabolism, and cell cycle control. A systematic analysis of the interaction between a large number of proteins in biological systems is required to understand the working principle of proteins in biological systems, the reaction mechanism of biological signals, and energy metabolism under special physiological states such as diseases, as well as the functional relationships between proteins. The STRING database (Szklarczyk et al., 2019) is a database that searches for interactions between known and predicted proteins. The database containins 9.6 million proteins and 1.38 million protein-protein interactions from 2,031 species. It contains results obtained from experimental data, results mined from PubMed abstract text, and combining results from other databases as well as results predicted using bioinformatics methods. We constructed protein-protein interaction networks for RA prognosis-related differentially expressed genes and glioblastoma-related differentially expressed prognostic genes respectively using the STRING database.

### Construction of Hub Gene-miRNA and Hub Gene-Transcription Factor Interaction Networks

In the post-transcriptional stage, the interaction with target genes by miRNA or TF control gene expression under disease-limiting conditions was analyzed (Baldwin 2001; Soifer et al., 2007). We obtained miRNAs and transcription factors of the differentially expressed genes associated with rheumatoid arthritis. The differential expression gene-miRNA network and differential expression prognostic gene-TF network associated with rheumatoid arthritis were visualized using Cytoscape software.

### Functional Enrichment Analysis

Gene ontology (GO) (2015) functional annotation analysis, which includes biological process (BP), molecular function (MF), and Cellular Component (CC), is a common method for large-scale gene functional enrichment. The Kyoto Encyclopedia of Genes and Genomes (KEGG) (Kanehisa et al., 2017) is a polular database that stores information about genomes, biological pathways, diseases, and medications. The ClusterProfiler package (Yu et al., 2012) was implemented in R for GO functional annotation analysis and KEGG pathway enrichment analysis of differentially expressed glioblastoma genes. In this study, pvalue<0.05 was considered statistically significant.

### GSEA Enrichment Analysis

GSEA (Gene Set Enrichment Analysis) was used to evaluate the distribution trend of genes in a predefined Gene Set in the Gene list sorted by phenotypic relevance, and thus to judge its contribution to phenotype (Subramanian et al., 2005). We obtained “C2. kegg.v7.4. symbols” and “c5. go.v7.4. symbols” gene sets from the MSigDB database for GSEA analysis of the two data sets respectively, and used the “ClusterProfiler” R package for GSEA analysis (Yu et al., 2012). Pvalue<0.05 was considered to be statistically significant (Liberzon et al., 2015).

### Statistical Analysis

R programming (https://www.r-project.org/, version 4.0.2) was used to execute all data processing and statistical. To compare two continuous variable groups, the statistical significance of the normally distributed variables was evaluated using the independent Student t-test, and the differences between the non-normally distributed variables were analyzed using the Mann-Whitney U test (i.e. Wilcoxon rank-sum test). *p* <0.05 was considered statistically significant when all statistical *p* values were bilateral.

## Results

### Overall Process of Experimental Design

**FIGURE 1:**
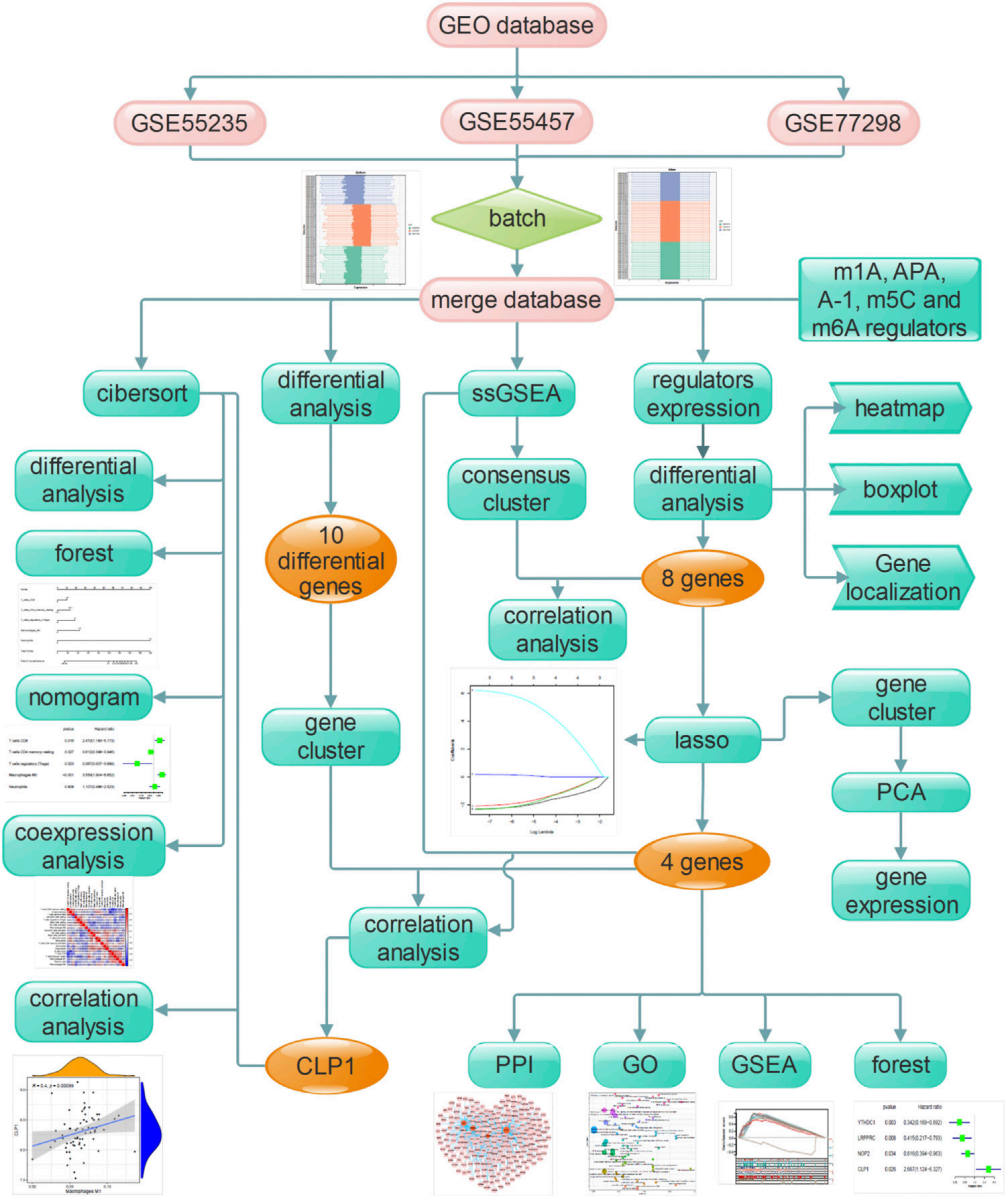
The flow chart of the current study. This study compared the expression characteristics of RNA modifiers and immune infiltration characteristics in rheumatoid customs and normal synovial tissues, constructed a prediction model and a miRNA/transcription factor-RNA modifier interaction network, and performed molecular subtype analysis to screen out the diagnosis landmark.

The flow chart designed in this study is shown in Figure 1. In short, we compared the expression characteristics of RNA modification factors in rheumatoid arthritis and normal synovial tissues by screening the expression matrix of samples from the GEO database. Then CIBERSORT was used to identify rheumatoid arthritis immune cell infiltration. Next, LASSO was used to identify key genes and immune cells for functional analysis. Molecular typing was performed according to the expression pattern of RNA modification factors, and protein interaction network analysis was performed to construct miRNA/transcription factor-RNA modification factor interaction network. Finally, correlation analysis was conducted between screened diagnostic markers and molecular subtypes and immune cells.

### Analysis of Overall Expression Characteristics of RNA Modification Factors in Synovial Tissues of Rheumatoid Joints

**FIGURE 2:**
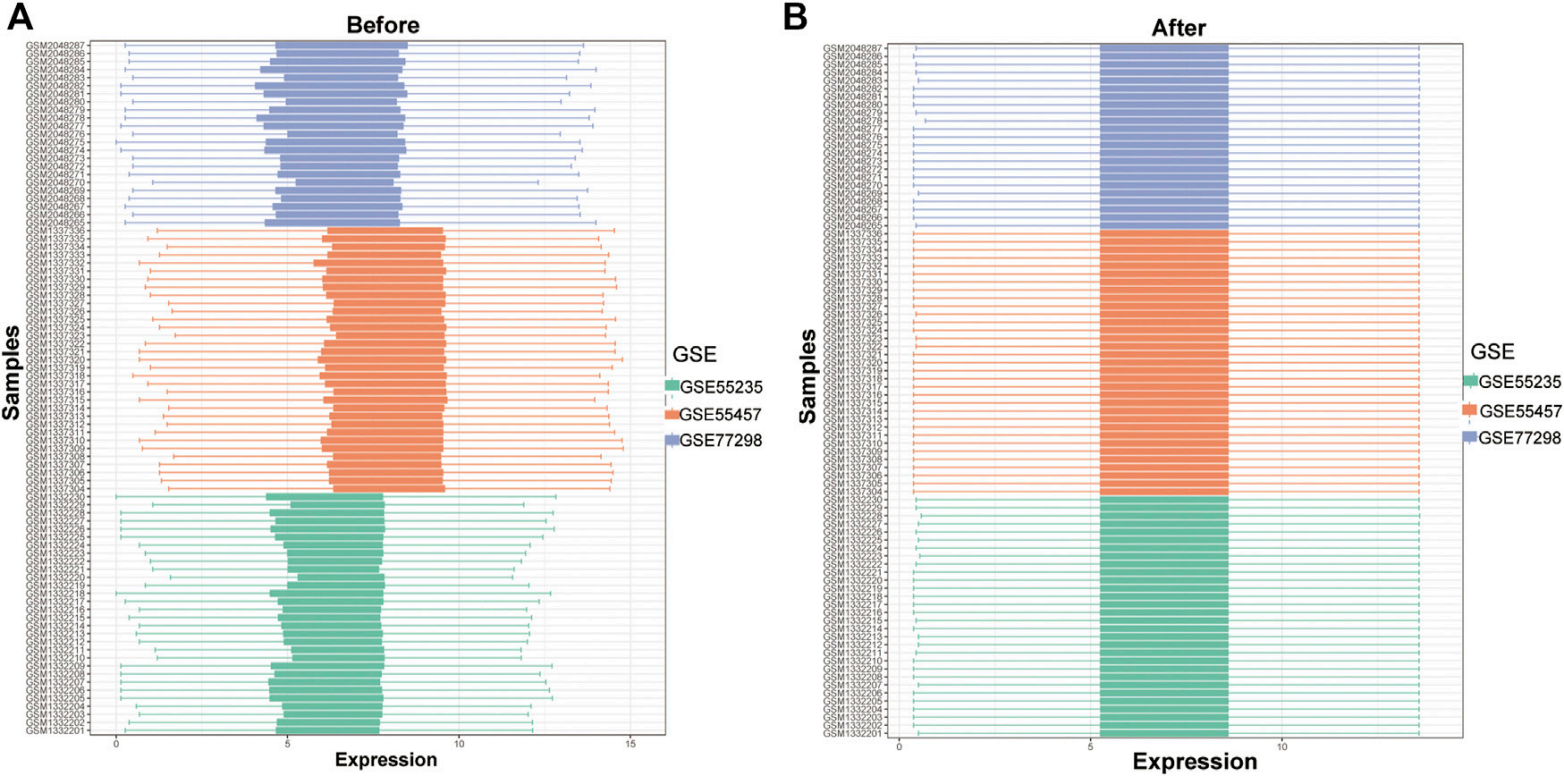
Data integration of typhoon expression matrix. **(A)**: The overall gene expression values of the three GEO data sets before correction, **(B)**: The overall gene expression values of the three GEO data sets after correction.

**FIGURE 3:**
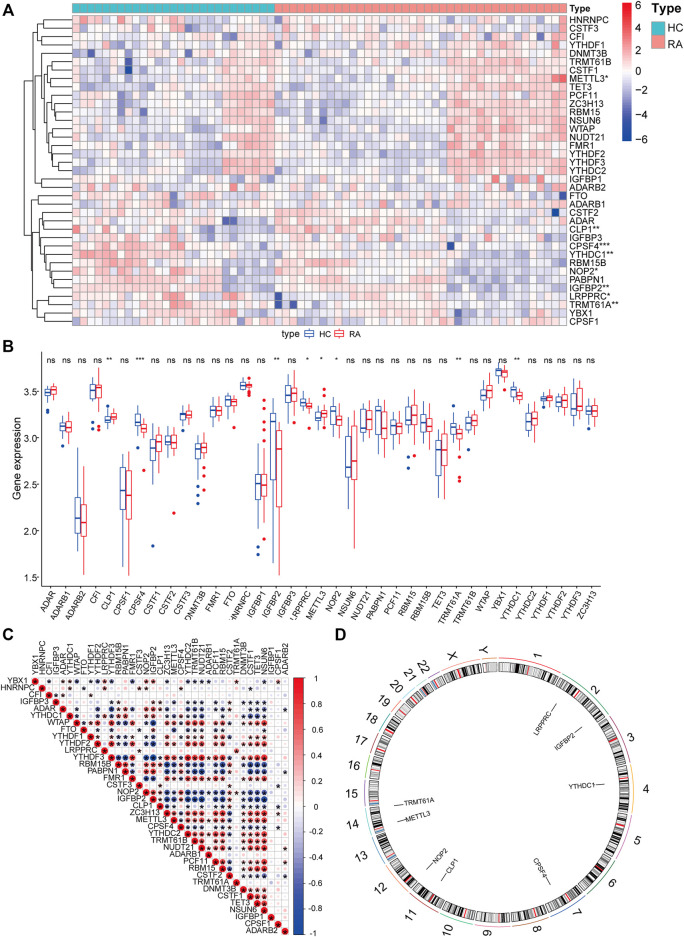
Expression characteristics and gene location of RNA modifiers. **(A)**: Heat map shows the expression characteristics of RNA modifiers in rheumatoid synovial tissues and normal tissues. Red stands for high expression level, and blue for low expression level; **(B)**: box plot shows the difference in the expression of RNA modifiers in synovial tissues and normal tissues, with significant differences in the expression of 8 genes. **(C)**: Correlation analysis of RNA modifiers, positive correlation is represented by red while negative correlation is represented by blue. **(D)**: The position of a differential gene on the chromosome (All figures * represents *p* <0.05, ** represents *p* <0.01, *** represents *p* <0.001)

To analyze the influence of gene expression values on the synovial tissues of rheumatoid joints relative to normal tissues, we first performed differential gene expression analysis on the integrated gene expression matrix using the limma package (Figure 2). 950 differential genes were identified in the comparison between the synovial tissues of rheumatoid joints and normal synovial tissues. There were 427 up-regulated genes and 523 down-regulated genes. We further observed the influence of RNA modification factors on synovial tissues of rheumatoid arthritis. The heat map showed that the expression of most RNA modification factors was relatively low in rheumatoid joint tissues (Figure 3A), and a total of 8 modification factors were significantly different from normal tissues: The expression of CPSF4, IGFBP2, LRPPRC, METTL3, NOP2, TRMT61A, and YTHDC1 was significantly decreased in rheumatoid joints, while the expression of CLP1 was significantly increased (Figure 3B). The expression of a significant number of RNA modification factors was correlated, according to the correlation analysis results. For example, YTHDC1 expression was significantly positively correlated with YBX1 and HNRNPC expression, while CPSF4 expression was significantly positively correlated with YTHDF3 expression (Figure 3C). Chromosome location showed that LRPPRC and IGFBP2 genes were located on chromosome 2. YTHDC1 is located on chromosome 4, CPSF4 on chromosome 7, CLP1 on chromosome 11, NOP2 on chromosome 12, METTL3, and TRMT61A on chromosome 14 (Figure 3D).

### Immune Infiltration in Rheumatoid Arthritis

**FIGURE 4:**
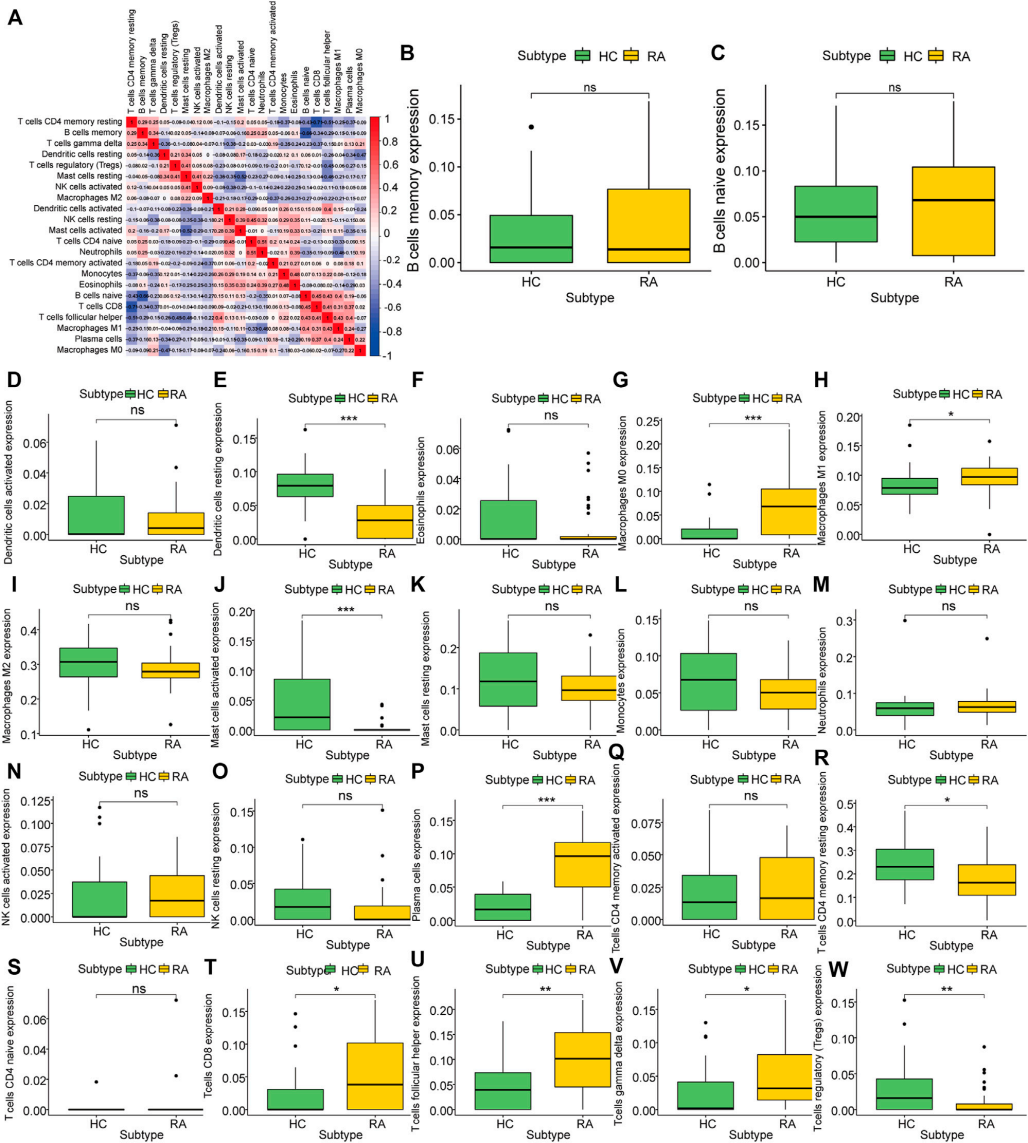
Characteristics of Rheumatoid Arthritis Immune Infiltration. **(A)**: Correlation of infiltration degree of 22 kinds of immune cell in synovial tissue, **(B–W)**: Difference analysis of immune cell infiltration degree between synovial tissue and normal tissue in rheumatoid arthritis.

To analyze the difference in the degree of immune infiltration between rheumatoid arthritis synovial tissue and normal tissue, we used the cibersort algorithm to calculate the degree of infiltration of 22 kinds of immune cells in rheumatoid arthritis tissue and normal tissue. Correlation analysis showed that there was a correlation between the degree of cell infiltration. For example, there was a positive correlation between B cells naïve and CD8+T cell infiltration (correlation coefficient was 0.48, Figure 4A). Comparing RA samples and normal tissue samples, we found that the average infiltration levels of dendritic cells resting, mast cells, T cells CD4 memory resting, Tregs and other cells in RA tissues were significantly lower than those in the healthy group, while M0/M1 macrophages, plasma cells, CD8 T cells, T cells follicular helper and T cells gamma delta were significantly higher than normal samples (Figure 4B–W) using the Wilcox. test algorithm. It is not difficult to see that these differentially infiltrated cells have more T cell subsets, and include three different subsets of macrophages, indicating that there may be a complex interaction between them.

## Molecular Clusters of RNA Modification Factors

**FIGURE 5:**
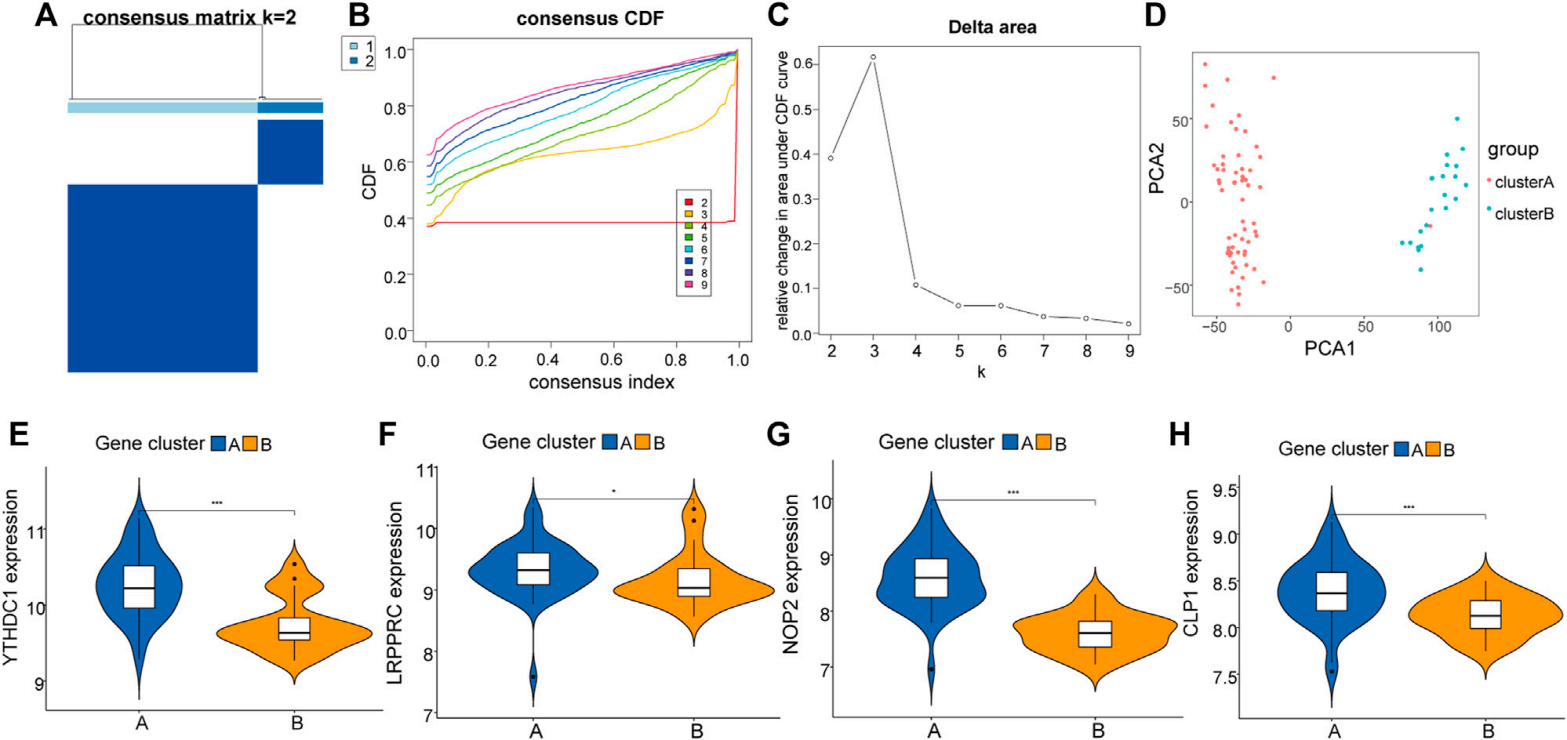
Molecular cluster of RNA modifiers. **(A–C)**: Clustering of synovial samples based on RNA modifiers. **(D)**: PCA analysis under different groups, where red is cluster A and blue is cluster B. **(E–H)**: Differences in the expression of hub genes under different groups of.

To further explore the biological characteristics of the expression of RNA modification factors in different synovial tissues, we used 8 RNA modification factors expression pairs to perform unsupervised consensus clustering of synovial tissues. The optimal separation was obtained by dividing all samples into two different subtypes (A: *n* = 45; B: *n* = 21, Figures 5A–C). Principal component analysis (PCA) results showed high separation quality (Figure 5D). Further differential analysis revealed that the expression of YTHDC1, LRPPRC, NOP2, and CLP1 in various subgroups differed significantly (Figures 5E–H). Therefore, we defined these four genes as hub genes.

## Construction of Rheumatoid Arthritis Prediction Model

**FIGURE 6:**
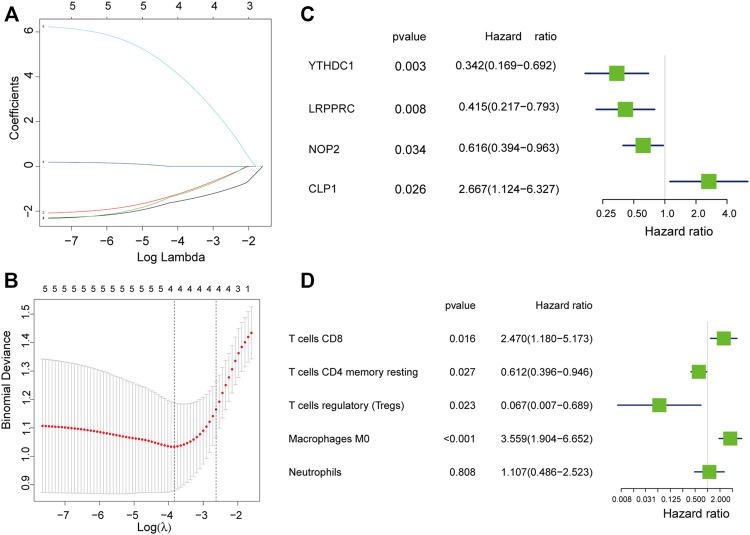
Model construction of hub gene and immune cell. **(A,B)**: Determine the best penalty value in the LASSO regression algorithm, and screen the RNA modifiers and immune cells most related to rheumatoid arthritis. **(C,D)**: Uses forest plots to display the screened RNA modifiers and immune cells.

**FIGURE 7:**
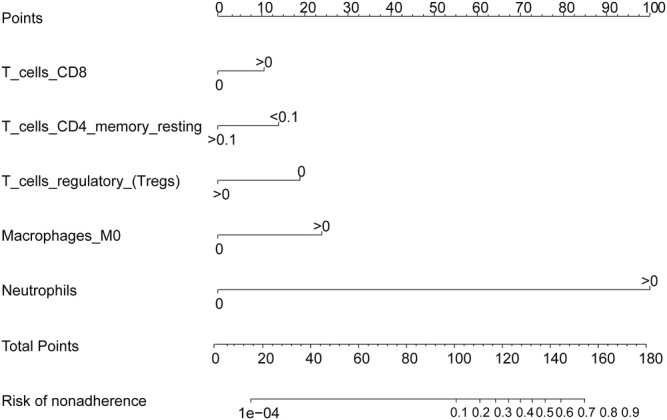
Immune cell prediction model nomogram. The nomogram was constructed using the immune cell prediction model.

We used the LASSO regression algorithm to construct a prediction model of rheumatoid arthritis based on RNA modification factors and immune cell infiltration levels (Figures 6A,B). The results showed that we constructed predictive models for the expression of YTHDC1, LRPPRC, NOP2, and CLP1 respectively and the prediction models of five immune cells: T cells CD8, T cells CD4 memory resting, T cells regulatory (Tregs), Macrophages M0, and Neutrophils (Figures 6C,D). Among them, CLP1 gene and Tregs immune cells were the most influential factors in the model. The results of the immune cell prediction model are presented in the form of a nomogram (Figure 7).

## Related Genes and Functional Analysis of Hub RNA Modifiers

**FIGURE 8:**
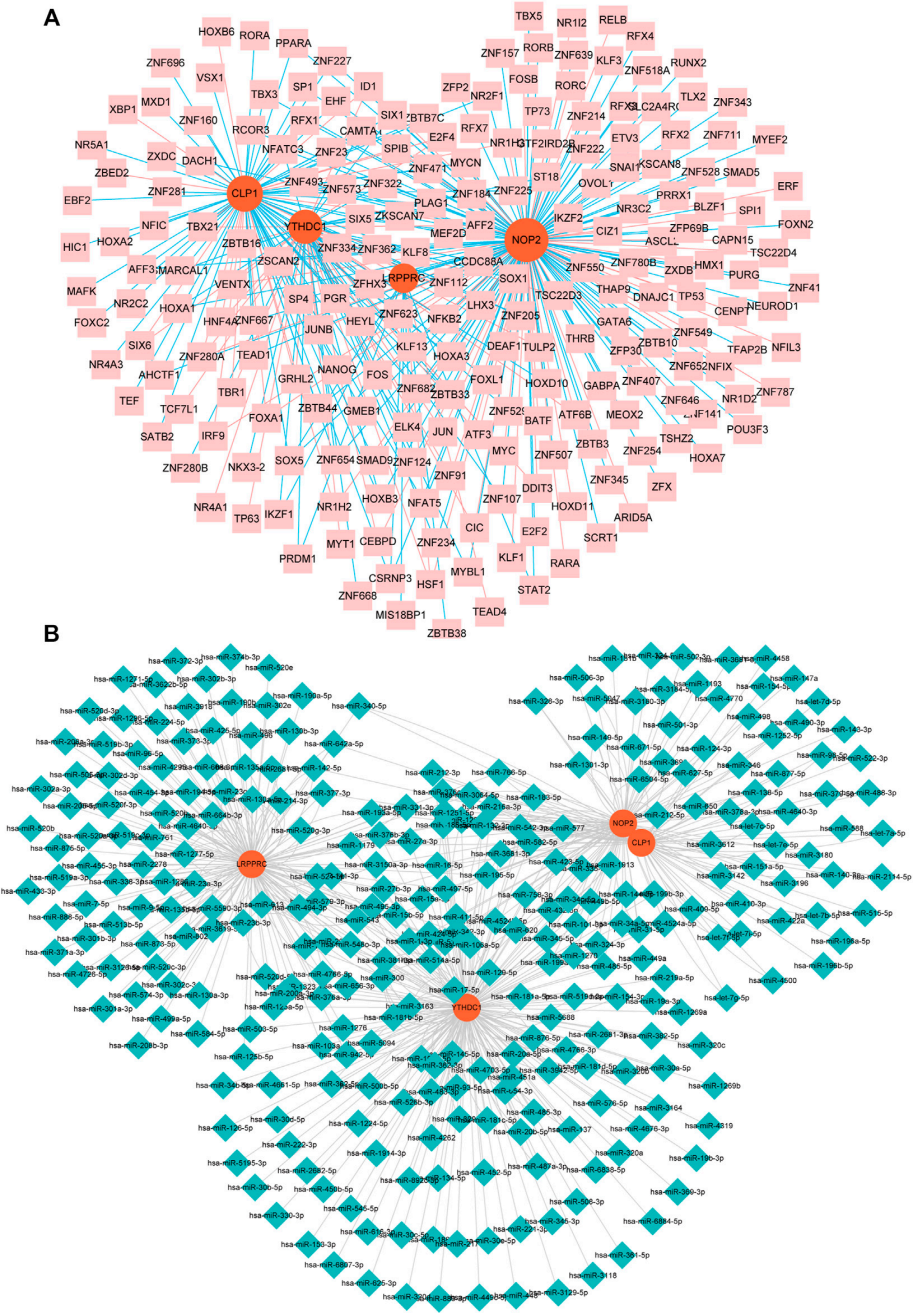
miRNA- and TF-RNA modifier network construction. **(A)**: mRNA-TF network of hub genes related to RA, pink nodes indicate TF, red nodes indicate key genes related to RA. **(B)**: Keys related to RA in the mRNA-miRNA network of genes, the blue nodes represent miRNAs, and the red nodes represent key genes related to RA.

We constructed an mRNA-TF network of differentially expressed genes related to rheumatoid arthritis, which contained 4 mRNAs and 218 TFS (Figure 8A). Among them, single TF targets at most 3 RNA modification factors at the same time, and there are 17 such TF, including AFF2, CAMTA1, CSRNP3, E2F4, ELK4, HOXB3, HSF1, ID1, KLF13, NFATC3, SOX5, ZBTB33, ZNF124, ZNF205, ZNF280A, ZNF654, and ZSCAN2, additionally, the modifier NOP2 was regulated by the most TF (177 in total), followed by CLP1 (86 in total).

### Related Genes and Functional Analysis of Hub RNA Modifiers

We constructed an mRNA-miRNA network with differential expression of RNA modifiers associated with rheumatoid arthritis, which contained 4 mRNA and 299 miRNAs (Figure 8B). Among them, the first seven miRNAs that simultaneously control multiple rheumatoid arthritis-related differentially expressed prognostic genes are: hsa-miR-494-3p controlling 9, hsa-miR-381-3p controlling 8, and hsa-miR-300 controlling 8 while Hsa-mir-376a-3p, HSA-Mir-3681-3p, HSA-Mir-432-5p, and HSA-Mir-543 respectively controlling 1 differentially expressed prognostic genes associated with RA.

Notably, in the mRNA-TF interaction network, TFs associated with key mRNAs are related to the progression of RA in several studies. For example, Ling-Hua Chang et al. found that after knocking out CEBPD, a protein commonly highly expressed in RA, joint damage in mice with collagen-induced arthritis was significantly lower than that in wild-type mice (Chang et al., 2012). Similar studies by Takeo Isozaki et al. pointed out that the expression of LD1 is highly correlated with CXCL16 in RA and is an essential factor affecting the inflammatory response of RA and the formation of synovial pannus (Isozaki et al., 2014). In addition, some miRNAs interacting with key mRNAs, such as miR-410-3p (Wang et al., 2020), miR-140-3p (Luo et al., 2021), etc., also constitute a vital part of the regulation of the RA process. These findings and our conclusions mutually support each other, revealing new possibilities for the treatment of RA.

**FIGURE 9:**
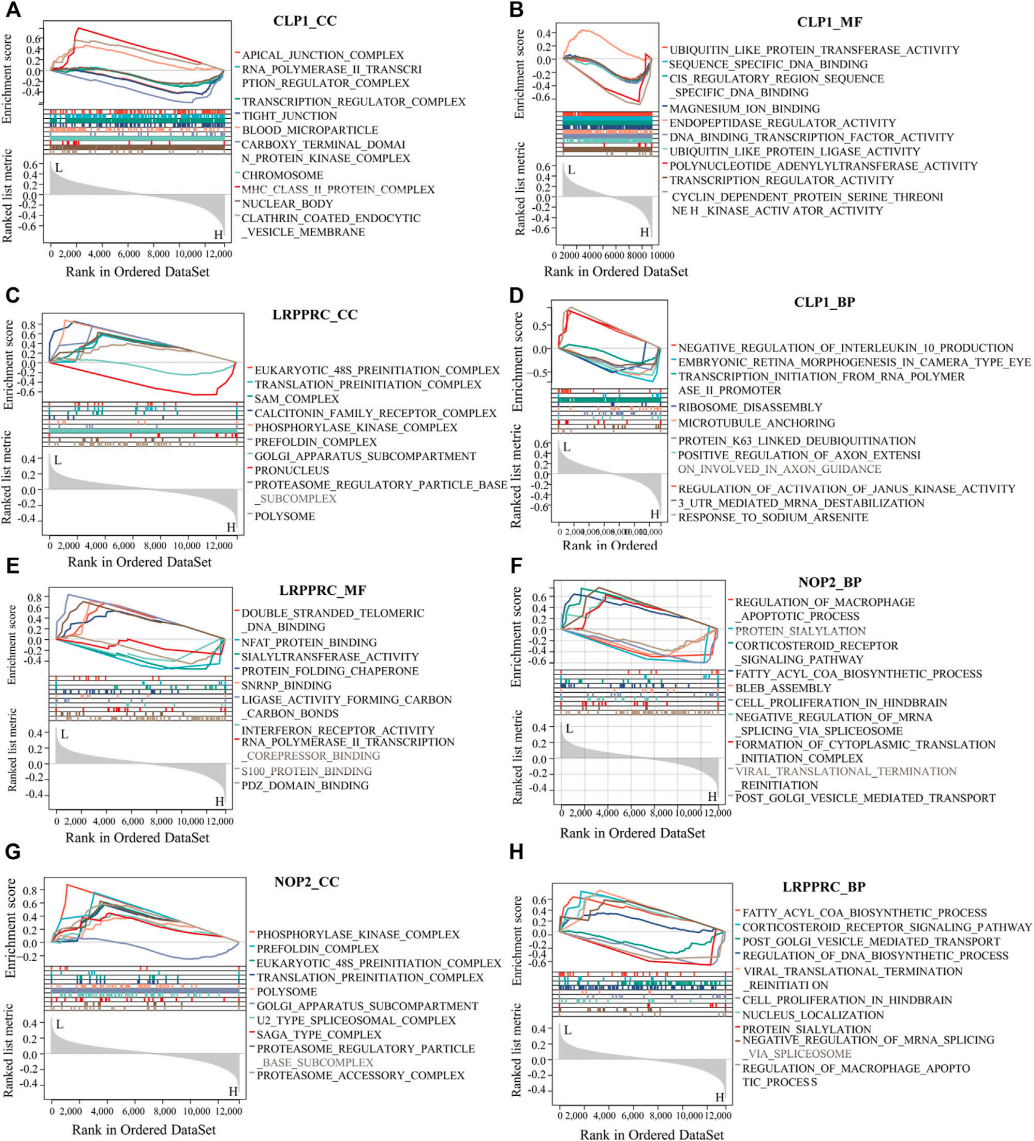
GSEA-GO analysis of key genes. **(A–H)**: The results of functional enrichment of GSEA-GO (including BP, CC, and MF) showing CLP1, YTHDC1, LRPPRC, and NOP2 respectively displayed.

**FIGURE 10:**
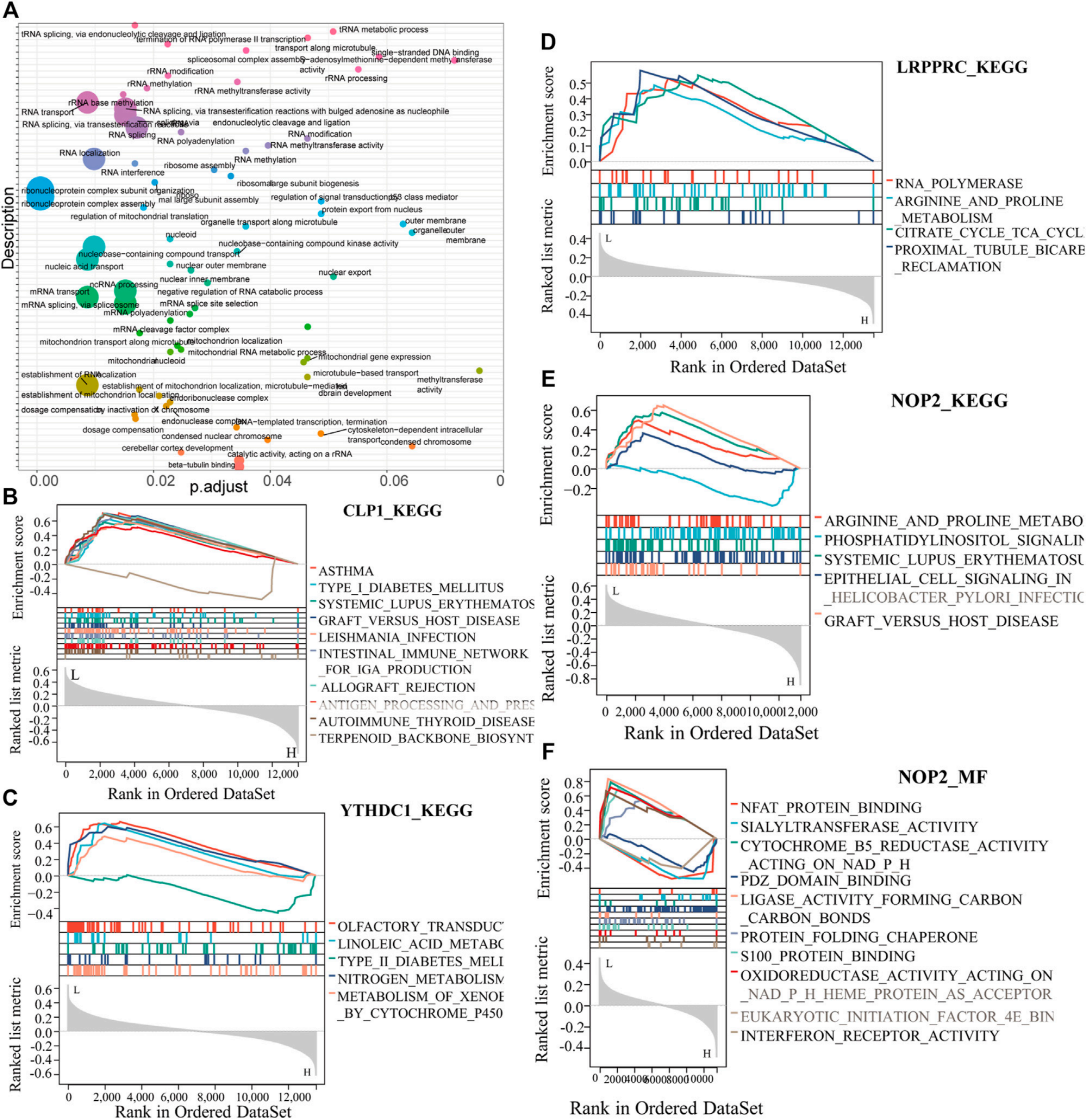
Key gene GESA-KEGG analysis. **(A)**: Summary of key gene GSEA-KEGG functions, the horizontal axis represents the P-adjust value, **(B–E)**: CLP1, YTHDC1, LRPPRC, NOP2 GSEA-KEGG function enrichment results. **(F)**: NOP2-MF function analysis.

To analyze the relationship between biological process, molecular function, cellular component, biological pathway and disease of the differentially expressed RNA modifiers related to rheumatoid arthritis, functional enrichment analysis of differentially expressed genes was first performed (Table 2). Further GSEA-GO analysis (Figure 9) showed that the differentially expressed genes associated with rheumatoid arthritis were also enriched in the regulation of macrophage apoptotic process, protein sialylation, corticosteroid receptor signaling pathway, post-Golgi vesicle mediated transport and other biological processes, phosphorylase kinase complex, eukaryotic 48s preinitiation complex, translation preinitiation complex, polysome, Golgi apparatus subcompartment, molecular functions such as NFAT protein binding, sialyltransferase activity, ligase activity forming carbon, carbon bonds, protein folding chaperone, s100 protein binding, interferon receptor activity in cell components. Next, GSEA-KEGG analysis was performed on DEGs, and the results are summarized in Figure 10A.

The results showed that it was enriched in arginine and proline metabolism, systemic lupus erythematosus, graft versus host disease, and other diseases (Figures 10B–F).

**FIGURE 11:**
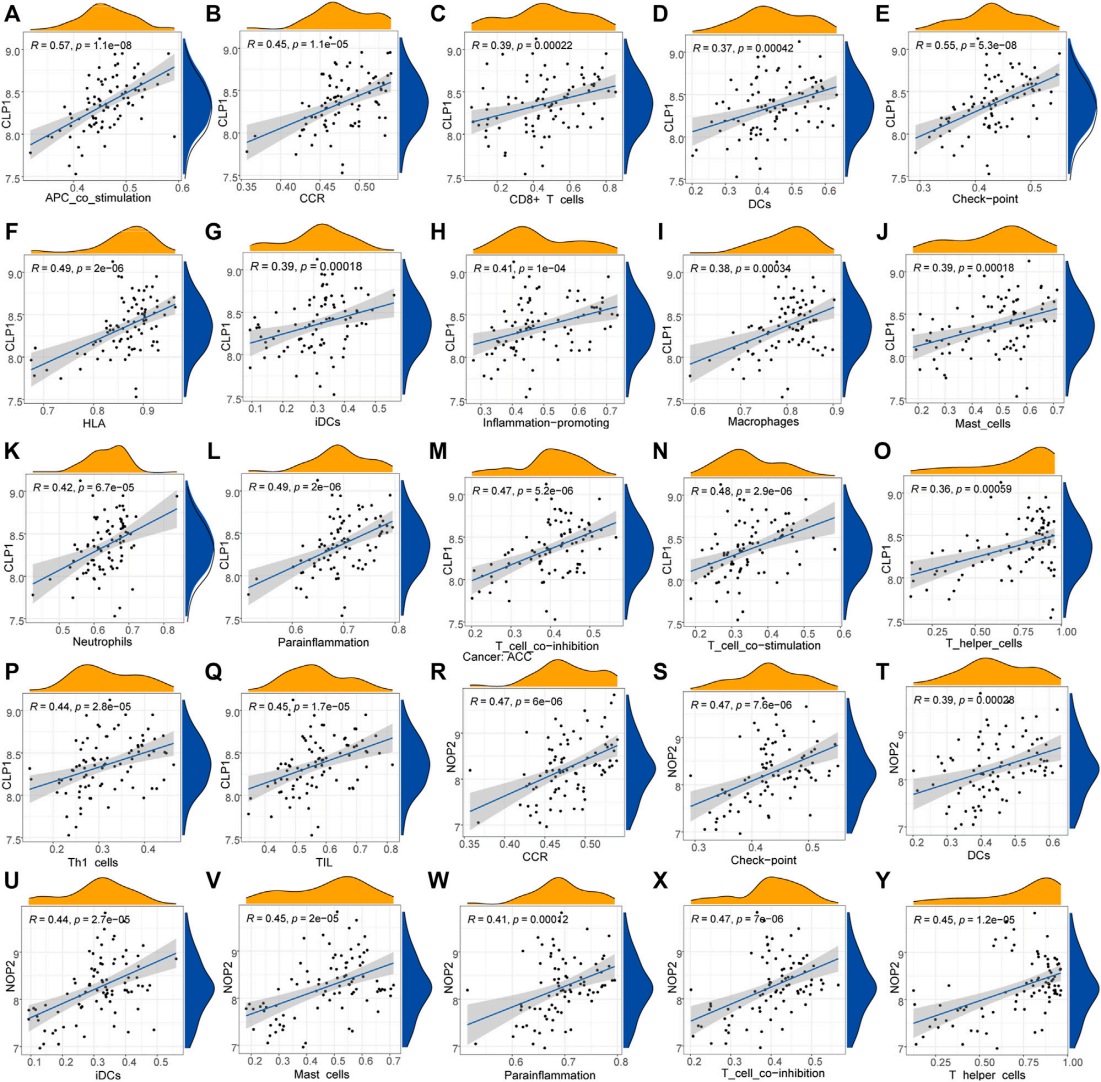
Correlation between key genes and immunity.

We further analyzed the correlation between key genes and the immune microenvironment. The expression of key genes CLP1 and NOP2 was significantly positively correlated with multiple immune indexes such as APC_co_stimulation,HLA,Parainflammation and the infiltration degree of various innate and acquired immune cells including CD8^+^ T cells,T helper cells, and mast cells, according to the correlation analysis of various immune indicators calculated by ssGSEA and hub gene expression (Figures 11A–Y.

A–Q: Correlation analysis between NOP2 and immune cells: the horizontal axis is correlation size, the vertical axis is significantly correlated immune cells, node size represents correlation strength, node color represents significance level. R-Y: Correlation analysis between CLP1 and immune cells. The horizontal axis is correlation size, the vertical axis is significantly correlated immune cells, node size represents correlation strength, and node color represents significance level.

### Molecular Clusters of Differential Genes in Rheumatoid Arthritis

**FIGURE 12:**
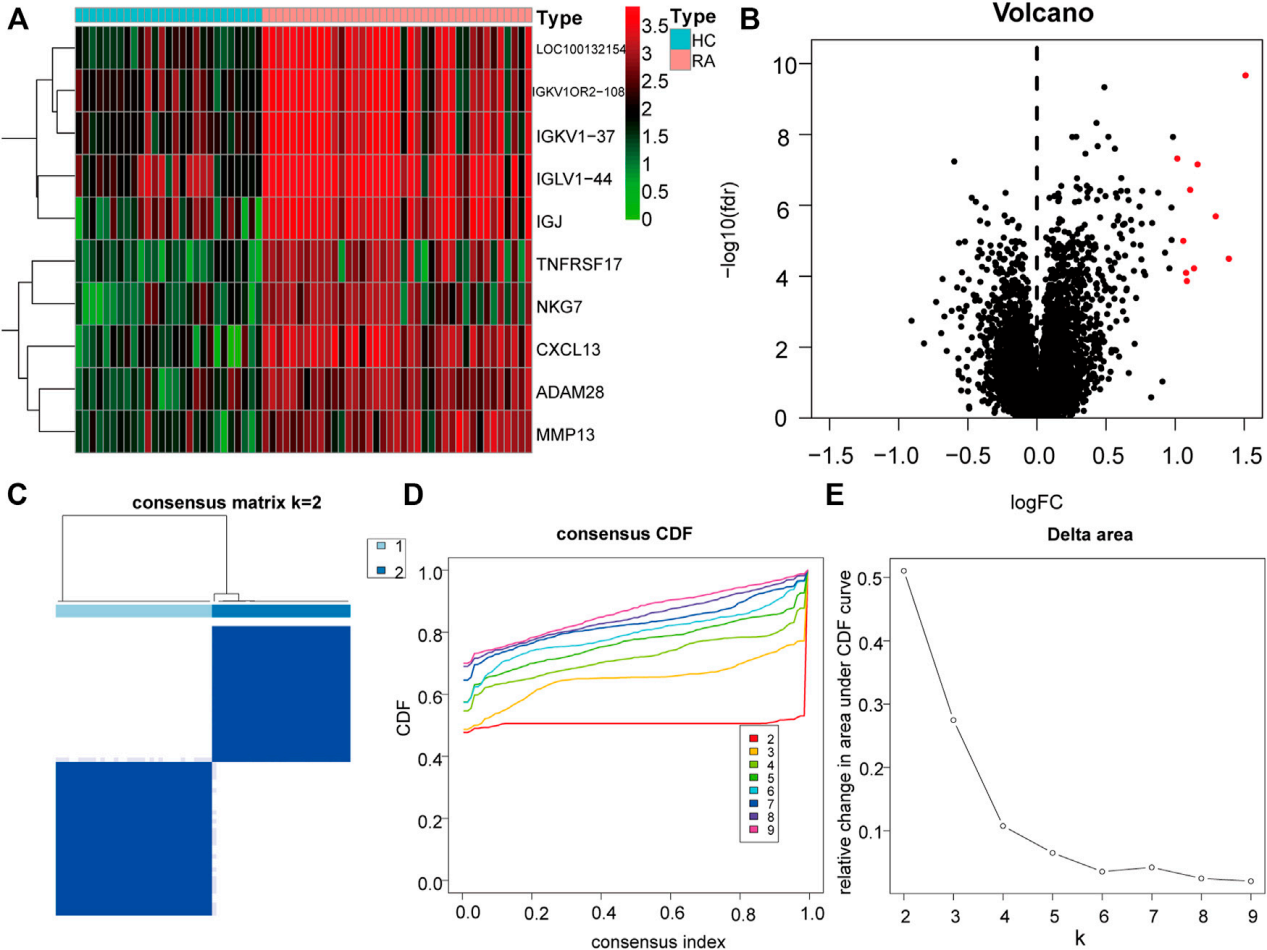
Differential Genes and Molecular Types of Rheumatoid Arthritis. **(A)**: The heat map depicts the differential genes between RA and normal synovial tissues, the top ten highly expressed genes in RA were selected, **(B)**: The volcano map depicts the differences in gene expression between RA and normal synovial tissues, and the top ten highly expressed genes in RA tissues in the category were chosen. **(C–E)**: Clustering and grouping of synovial samples based on differential genes for rheumatoid arthritis. **(D)**: PCA analysis under different groups, where red is cluster A and blue is cluster B. **(E)**: Differences in the expression of key genes in different groups.

**FIGURE 13:**
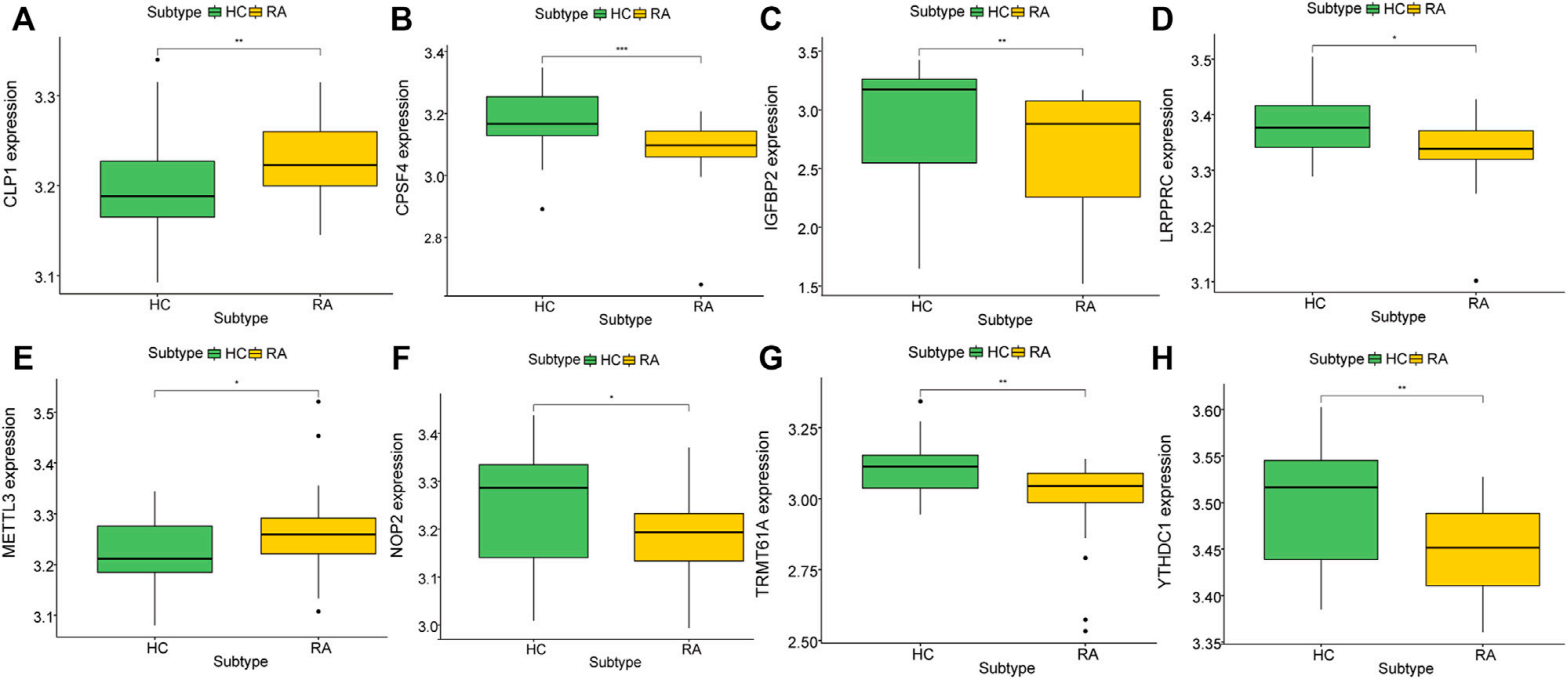
Correlation between key genes and molecular typing of rheumatoid arthritis. **(A–H)**: The expression of different rheumatoid arthritis differential genes and hub genes differed under molecular classification groupings, only CLP1 showed significant differences, thus we define it as a diagnostic marker.

To further explore the biological characteristics of gene expression in the synovial tissues of rheumatoid arthritis, we used the expression of differential genes in rheumatoid arthritis (Figures 12A,B) to conduct unsupervised consistent clustering again on synovial tissues. Optimal separation was achieved when all samples were divided into two different subtypes (I: *n* = 27; II: *n* = 39, Figures 12C–E). The expression of CLP1 was significantly different among different groups (Figures 13A–H), Therefore, we defined these genes as a diagnostic marker.

### Diagnostic Marker Analysis

**FIGURE 14:**
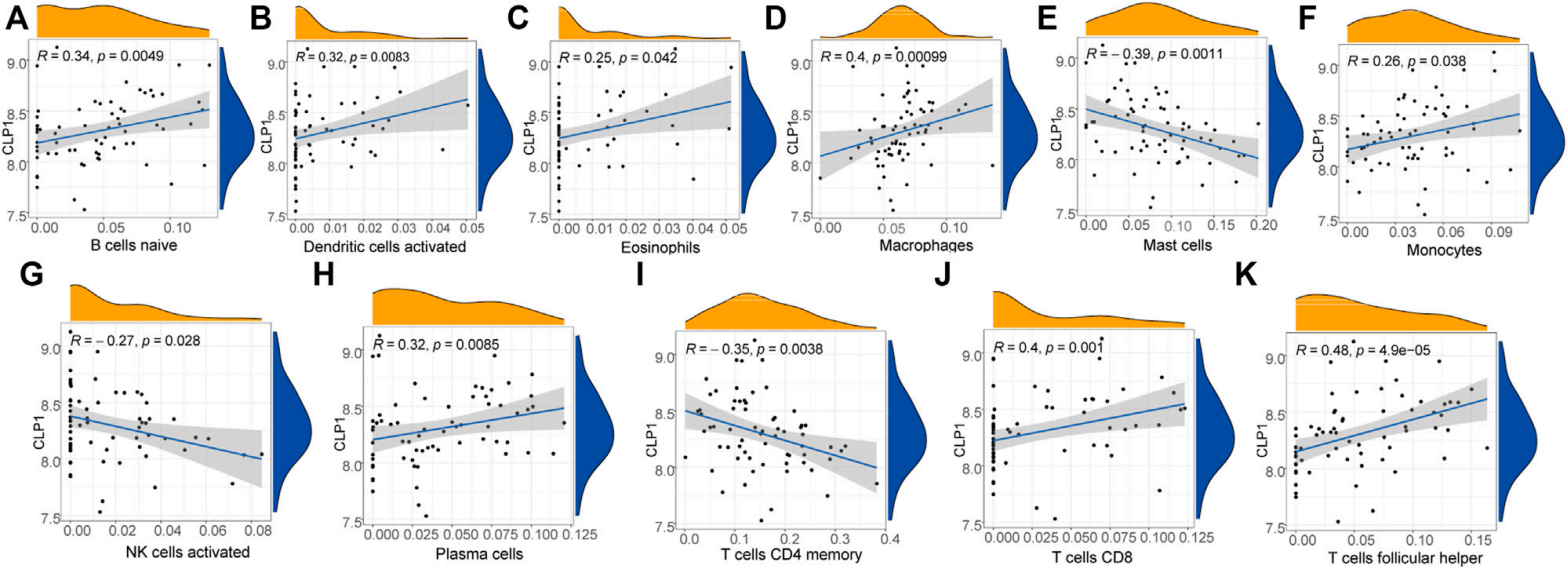
Diagnostic marker gene and immune correlation analysis. **(A–K)**: CLP1 gene correlation analysis with immune cells, the slope is the correlation size, and the Pvalue represents the significance level.

The immune correlation of diagnostic marker CLP1 was investigated, and the result demonstrated a significant positive correlation between the infiltration degree of B cells naïve, Dendritic cells activated, Eosinophils,Macrophages, Monocytes, Plasma cells,T cells CD8, and T cells follicular helper. of which, T cells follicular helper had the highest positive correlation (0.48), while mast cells, NK cells activated, and T cells CD4 memory infiltration revealed a significant negative correlation, with Mast cells having the highest negative correlation (-0.39) (Figure 14).

## Discussion

RA is one of the most common autoimmune diseases. Current research suggests that the destruction of synovial cartilage and bone caused by immune cell infiltration plays a pivotal role in the occurrence and development of RA (Coutant and Miossec,2020). As an important post-transcriptional regulator, RNA modification participates in the biological processes of a variety of eukaryotes, profoundly affects the development of organisms, and plays a key regulatory role in a variety of diseases (Su and Randau 2011; Meyer and Jaffrey 2014). Nevertheless, the mechanism of immune cell infiltration and RNA modification in RA has not been fully elucidated. In addition, the relationship between the two in the occurrence and development of RA has not yet been clarified. The purpose of our research is to explore the significance of RNA modification regulators in RA. The main goal of this research is to explore the relationship between RA and RNA modification and immune cell infiltration and to find clinically applicable serum markers.

In our research, 8 RNA modification regulators with significant differences in expression between RA synovial tissue and normal tissue were initially found from 5 RNA modification regulator gene sets, and 10 abnormal immune infiltrations in RA synovial tissue of immune cells were identified as well. On this basis, we identified 4 hub RNA modification regulator genes (YTHDC1, LRPPRC, NOP2, and CLP1) through cluster analysis. Combined with the analysis of the level of immune cell infiltration, the hub genes and infiltrating immune cell-related RA prediction model were established respectively, in which CLP1 gene and Tregs cells had the highest influence weights in the two models individually. CLP1 exhibits evident differential expression as shown by the molecular subtype analysis of differentially expressed genes here, hence we define CLP1 as a diagnostic marker for RA. In addition, we developed a nomogram based on 5 immune cells (T cells CD8, T cells CD4 memory resting, T cells regulatory (Tregs), macrophages M0, neutrophils) that can be used to predict the prognosis of RA patients. Furthermore, we also explored the interaction between the four key RNA modifiers and miRNA and TF. GSEA-GO and KEGG-GSEA analysis of hub genes were conducted, and further exploration of the relationship between the diagnostic marker CLP1 and immune cell infiltration was progressed.

CLP1 is one of the constituent proteins of the cleavage factor protein complex. It participates in the cleavage of the 3′untranslated region of newly synthesized mRNA molecules in the process of gene transcription. It is one of the post-transcriptional modifications necessary to produce mature mRNA (Hardy and Norbury 2016) (Ustyantsev et al., 2017). In addition, CLP1 is also involved in the precursor tRNA splicing process and plays an important role in the tRNA splicing endonuclease (TSEN) complex (Schaffer et al., 2014). In addition, Hiroyuki Fujinami et al. pointed out that CLP1 is the main RNA kinase in mice and is mainly used to phosphorylate the 5′end of RNA in the siRNA pathway (Fujinami et al., 2020). In disease research, Caitlin E Monaghan et al. found that homozygous mutations in CLP1 can affect the processing of mRNA 3′and ultimately lead to neurodegenerative disease, namely pontocerebellar hypoplasia type 10 (Monaghan et al., 2021); while Kitti Szoták-Ajtay et al. found that CLP1 knockout mice have abnormal lung expansion function, and Clp1 ^*K/K*^ embryos in late pregnancy showed impaired prenatal lymphatic function and impaired lung expansion (Szoták-Ajtay et al., 2020). However, it is worth noting that the existing relationship between CLP1 and immune cells is unclear. The study by Clotilde Guyon et al. pointed out that CLP1-mediated 3′UTR shortening may be involved in the expression of thymic medullary epithelial cells and the process of antigen presentation, thereby affecting the occurrence and development of autoimmune diseases (Guyon et al., 2020). The research of Kitti Szoták-Ajta and Clotilde Guyon confirmed the feasibility of our choice of CLP1 as a diagnostic indicator of RA to a certain extent.

RA is a chronic autoimmune illness characterized by persistent inflammation that has historically hampered therapeutic management (O'Neil and Kaplan 2019). Although the immunization research for RA continues to deepen, the RA-related immunization manner is still unclear. In our research, Tregs cells are considered to play an important role in the occurrence and development of RA. Existing studies believe that Tregs cells can alleviate the progression of RA by inhibiting a variety of inflammatory cytokines produced by the synovium of bones and joints (Shi and Chi 2019; Sjaastad et al., 2021); and in the hypoxic environment of synovium, T cells will be induced to differentiate by synovial fibroblasts. This leads to a decrease in the number of Tregs cells and an increase in the number of Th17 cells, which aggravates the progression of RA (Ding et al., 2020). After analyzing the correlation between CLP1 and immune cells, we found that CLP1 was highly positively correlated with T cells follicular helper (Tfh) infiltration, but was highly negatively correlated with mast cells expression. Recent research has discovered that uncontrolled Tfh cell expansion can be seen in a variety of systemic autoimmune disorders (Yao et al., 2021). the number of circulating Tfh-like (cTfh-like) cells, their subtypes, and synovial infiltrating T helper cell is linked to RA patients’ disease activity (Lu et al., 2021). The involvement of mast cells in the development of RA is currently unknown. In different reports, mast cells have two contradictory effects: pro-inflammatory and anti-inflammatory. For example, in the animal model of mast cell defect, the model animal shows resistance to arthritis (Pitman et al., 2011); in the same way, a recent study by Julio Ramı´rez et al. found that the density of mast cells was increased in 23 RA patients undergoing joint synovial tissue biopsy (Kim et al., 2021). On the contrary, the mRNA levels of synovial mast cell-specific genes in naive early RA patients with DMARD are negatively correlated with the severity of the disease (Rivellese et al., 2015). In addition, the levels of serum trypsin and synovial mRNA are negatively correlated with systemic inflammation through the detection of CRP levels (Rossini et al., 2016). The results of our study tend to show the negative correlation effect of mast cells infiltration in RA, which may be related to the heterogeneity of RA itself. The upregulation of CLP1 may affect mast cells in RA synovium through some unknown pathways which needs further study.

In the subsequent construction of the miRNA/TF-mRNA network, we found some miRNAs that regulate CLP1 are associated with RA. Interestingly, the role of these miRNAs in the pathogenesis of RA has not yet been studied. In addition, we have also noticed that there is a wide-ranging relationship between the modifier NOP2 and TF, and the role of this regulatory factor in RA is not yet clear. In addition, the results of GSEA-GO showed that CLP1 is also involved in the apoptosis process of macrophages. Studies have pointed out that the death of activated macrophages is related to the pathogenesis of RA (Yang et al., 2020). According to the report, the anti-apoptotic ability of pro-inflammatory macrophages in the synovial fluid of RA patients is higher than that of anti-inflammatory macrophages (Balogh et al., 2018), but the role of CLP1 and macrophage apoptosis in RA has not been elucidated. The KEGG-GSEA analysis results suggest that CLP1 is also involved in arginine and proline metabolism, systemic lupus erythematosus, graft-versus-host disease, and other disease processes, which also confirms the role of CLP1 in autoimmune system diseases.

In addition to this evidence, a recent review by Jiajie Tu et al. also pointed out that the interaction of macrophages with T cell populations has significant implications for the progression of RA. In general, macrophages can recruit or differentiate T cells toward pro-inflammatory subtypes (e.g., toward Th17); in turn, different T cell types can shift the balance of monocyte/macrophage differentiation toward disruption of Osteocytes, aggravating joint damage, and prompt macrophages to secrete various cytokines. Our findings also reveal the significant infiltration changes of macrophages and T cell subsets in the process of RA, which adds favorable evidence for the search for RA immunotherapy targets (Tu et al., 2021).

Although this research investigated the manner of RNA modification and immune cell infiltration in RA, there are still several limitations in our study. Firstly, even if the reliability of bioinformatics technology is promoted rapidly, further studies and experiments should be conducted to verify the RNA modification manner in RA. Secondly, 3 datasets were introduced and 66 joint synovial tissue samples were included in this study, but other examples such as serum samples in RA are unavailable. On the other hand, the degree of RA in samples should be classified. With the improvement based on the limitations above, our study could be verified solidly. Nevertheless, our research indicated a potential diagnostic and prognostic biomarker, CLP1, in RA, which could be the new therapeutic target in the clinic. Otherwise, we reveal the relationships among RNA modification, immune cell infiltration, and RA, which could update the acknowledge of the advance and onset in RA.

## Conclusion

In summary, we selected 4 key RNA regulatory factors and 5 infiltrating immune cells to establish a prediction model and nomogram of RA in this study and selected CLP1 as an important diagnostic marker in RA. On this basis, we also discussed the role of Tregs, Tfh, and mast cells in the occurrence and development of RA, hoping to provide new ideas for better clinical diagnosis and treatment of RA.

## Data Availability

The original contributions presented in the study are included in the article/Supplementary Material, further inquiries can be directed to the corresponding authors.
